# Age‐Specific Differences in Inflammatory Biomarkers and Their Impact on Futile Recanalization After Mechanical Thrombectomy: An Inverse Probability Weighting Analysis

**DOI:** 10.1111/ene.70182

**Published:** 2025-05-12

**Authors:** Gabriele Prandin, Mariarosaria Valente, Liqun Zhang, Paresh Malhotra, Simona Sacco, Matteo Foschi, Raffaele Ornello, Edoardo Pirera, Francesco Toraldo, Domenico Maisano, Caterina Del Regno, Filippo Komauli, Adelaida Gartner Jaramillo, Hakam AL‐Karadsheh, Hamza Zahid, Piers Klein, Mohamad Abdalkader, Paolo Manganotti, Kyriakos Lobotesis, Thanh N. Nguyen, Soma Banerjee, Gian Luigi Gigli, Giovanni Merlino, Lucio D'Anna

**Affiliations:** ^1^ Clinical Unit of Neurology, Department of Medicine, Surgery and Health Sciences University Hospital and Health Services of Trieste, ASUGI, University of Trieste Trieste Italy; ^2^ Department of Brain Sciences Imperial College London London UK; ^3^ Stroke Unit Udine University Hospital Udine Italy; ^4^ Clinical Neurology Udine University Hospital and DMED, University of Udine Udine Italy; ^5^ Department of Neurology St George's University of London London UK; ^6^ Department of Biotechnological and Applied Clinical Sciences University of L'aquila L'Aquila Italy; ^7^ Internal Medicine and Stroke Care Ward, Department of Promoting Health, Maternal‐Infant, Excellence and Internal and Specialized Medicine (ProMISE) “G. D'alessandro” University of Palermo Palermo Italy; ^8^ Department of Neurology, Radiology Boston Medical Center Boston Massachusetts USA; ^9^ Neuroradiology, Department of Imaging Charing Cross Hospital, Imperial College London, NHS Healthcare Trust London UK; ^10^ Department of Stroke and Neuroscience Charing Cross Hospital, Imperial College Healthcare NHS Trust London UK

**Keywords:** futile recanalization, intravenous thrombolysis, mechanical thrombectomy

## Abstract

**Background:**

Mechanical thrombectomy (MT) is the standard treatment for large vessel occlusion (LVO) stroke. However, a substantial proportion of patients experience poor functional outcomes despite successful reperfusion, namely futile recanalization (FR). This study aimed to evaluate the predictive value of inflammatory biomarkers, measured on admission and at 24 h, in identifying the risk of FR and to assess age‐specific differences influencing this outcome.

**Methods:**

This international, multicenter, observational study included patients with anterior circulation LVO stroke treated with MT. Strict inclusion criteria were applied to minimize confounding factors related to inflammation. Inflammatory biomarkers were assessed at admission and 24 h post‐procedure. Inverse probability weighting (IPW) was utilized to balance baseline characteristics between patients with FR and effective recanalization (ER). Least absolute shrinkage and selection operator (LASSO) regression was applied to identify independent predictors, and restricted cubic splines were used to determine optimal biomarker cut‐offs.

**Results:**

Among 885 patients, 470 (53%) experienced FR. In multivariate analysis, 24‐h CRP (OR 1.01, 95% CI 1.01–1.02, *p* = 0.018) and 24‐h NLR (OR 1.11, 95% CI 1.02–1.22, *p* = 0.019) were significant predictors of FR, with cut‐offs of 8.55 and 4.58, respectively. In patients aged < 80 years, 24‐h CRP and NLR were most predictive (cut‐offs: 17.09 and 5.59). In patients aged ≥ 80 years, admission SIRI emerged as the most significant predictor (OR 1.24, 95% CI 1.06–1.50, *p* = 0.015), with an optimal cut‐off value of 2.53.

**Conclusions:**

Inflammatory biomarkers exhibit significant predictive value for FR following MT, with distinct age‐specific patterns. These findings underscore the importance of tailoring predictive models and interventions to optimize clinical outcomes.

## 1 Introduction

Mechanical thrombectomy (MT) is the standard of care for acute ischemic stroke due to large vessel occlusion (LVO), regardless of the age of the patients [1]. Elderly patients (aged ≥ 80 or older) account for a major proportion of ischemic strokes worldwide, and the use of MT in the elderly has been increasing in recent years [2]. Major clinical trials have demonstrated the benefit of EVT for the general population, although the degree of benefit is less clear in the elderly population, as this subgroup was often excluded or under‐represented in past trials [3, 4, 5, 6]. Previous studies have shown that a good functional outcome is achieved in only a small proportion of octogenarians undergoing MT for acute ischemic stroke, although the recanalization rates in this population are comparable to those observed in younger patients [7, 8]. Thus, a substantial proportion of patients aged ≥ 80 fail to achieve a good functional outcome at 90 days post‐MT despite successful recanalization, a phenomenon referred to as futile recanalization (FR) [2, 7].

Previous studies have explored the main predictors of FR following MT in acute ischemic stroke caused by LVO in the anterior circulation [9, 10, 11, 12, 13, 14, 15, 16, 17, 18]. However, data specifically addressing the role of inflammatory biomarkers stratified by age groups remain limited due to unspecified exclusion criteria and heterogeneous time points for blood test collection, which hinder the ability to draw consistent conclusions [10, 11, 12, 13, 14, 15, 16, 17]. The aim of this study is to evaluate the predictive role of inflammatory biomarkers, both on admission and at 24 h, for futile recanalization (FR) in a large, multicentre cohort of patients treated with MT. Additionally, we aimed to investigate age‐related differences across various age groups that may influence the risk of FR following MT.

## 2 Methods

### 2.1 Study Design and Patients

This was a multicentre, observational, investigator‐initiated, retrospective study that included all acute stroke patients aged 18 years or older consecutively treated with MT in four thrombectomy centers: Charing Cross Hospital, Imperial College Healthcare NHS Trust, London (UK); Udine University Hospital, Udine (Italy); St George's University of London, London (UK); and Boston Medical Center (USA) between January 1st, 2016 and 30th March 2023, with local stroke registries available [19, 20, 21, 22]. The study was conducted in accordance with the Declaration of Helsinki (1964 and subsequent revisions). This study follows the STROBE Checklist for observational studies [23]. This study has obtained approval from the UK Health Regulator Authority (Health Regulator Authority Reference No: 275260). The study has also received confirmation of capacity and capability from the Imperial College Healthcare NHS Trust.

### 2.2 Patient Inclusion and Exclusion Criteria for the Analysis

The criteria for patient selection were: (1) age ≥ 18 years; (2) National Institutes of Health Stroke Scale (NIHSS) score 6 or more; (3) Alberta Stroke Program Early CT score (ASPECTS) 5 or more; (4) LVO sites: distal internal carotid artery, middle cerebral artery segments M1 or M2; (5) initiation of the MT had to be possible within 6 h after the stroke onset; [6] pre‐event modified Rankin Scale (mRS) score of 0 to 2; Intravenous thrombolysis (IVT) with intravenous tissue plasminogen activator (tPA) was administered in all patients who presented within 4.5 h of stroke symptom onset and without contraindications according to the guidelines [23]. For this analysis, we excluded stroke patients with basilar artery occlusion and patients who met DAWN or DEFUSE 3 eligibility criteria [24, 25]. Moreover, for this analysis, we excluded patients with concomitant conditions which could potentially alter the inflammation biomarkers, in particular: (1) ongoing effect of immunomodulatory or immunosuppressive drugs, (2) ongoing infections or infections developed within 48 h after admission, (3) chronic inflammatory diseases, (4) hematological disorders, (5) cancer (active and/or under treatment), (6) major trauma or surgical procedures in the previous 28 days, (7) acute myocardial infarction (AMI) with or without ST elevation, (8) severe liver or kidney dysfunction (eGFR < 30 mL/min) as per literature references [26, 27] and (9) recent transfusion (< 7 days before admission) or transfusion done within 24 h after admission.

### 2.3 Data Collection

All information was collected prospectively, such as medical history, demographic characteristics, history of previous stroke or transient ischemic attack (TIA), baseline NIHSS, admission therapy, site of the occlusion, procedural management, and variables, and key time points. Two independent and trained raters for each center who did not participate in the endovascular stroke treatment of the included patients and were blind to any clinical and treatment information evaluated the modified Rankin Scale (mRS) of the patients at 90 days centrally through a telemedicine consultation or in‐person consultation. Any disagreement was resolved with the involvement of a senior stroke neurologist for each center as a third party not involved in the care of the patients. The investigators received training and qualification certificates to record the NIHSS and modified Rankin Scale (mRS). NIHSS was performed in all patients on admission and at 24 h after the MT. The prescription of any antiplatelets and anticoagulant before admission was recorded and included the use of any direct oral anticoagulant (DOAC) therapy (defined as one of the following drugs and dosages: apixaban 2.5 mg or 5 mg twice daily; dabigatran 110 mg or 150 mg twice daily; edoxaban 30 mg or 60 mg once daily; or rivaroxaban 15 mg or 20 mg once daily); vitamin K antagonist (VKA) (defined as treatment with acenocoumarol/warfarin). The choice of treatment was decided by the treating physician as part of routine clinical care pre‐admission. The extent of the initial core infarct was determined on pre‐therapeutic CT using ASPECTS. Revascularization was assessed by applying the modified thrombolysis in cerebral infarction (TICI) classification [28]. Successful recanalization was defined as grade 2b, 2c, or 3 of recanalization. Hemorrhagic transformation (HT) was also assessed: it was considered as HT if it was not seen on the admission brain scan with or without a decline in neurological status, including all the subtypes following the Heidelberg classification [29]. sICH was identified as any intracranial hemorrhage on neuroimaging with a ≥ 4 point increase of the NIHSS score from baseline [30]. Anesthesia was provided by neuroanesthesiologists who were present for all MT procedures as general anesthesia (GA) or local anesthesia (LA). For the purpose of this analysis, we considered GA patients those who initially underwent LA and then subsequently converted to GA. The participating centers were required to confirm and revise any missing, inconsistent, or extreme values. Frailty was assessed in patients aged 80 and above using the Clinical Frailty Scale (CFS) by Rockwood et al. (2020) [31].

### 2.4 Blood Samples

Venous blood samples were obtained from all patients at admission and after 24 h. Analyses of inflammatory markers—including WBC, neutrophil count (N), platelet count (P), lymphocyte count (L), and monocyte (M)—were conducted in the local laboratory department. Laboratory measurements were performed 60 min after the onset of blood collection. The NLR (Neutrophil to Lymphocyte ratio) was determined as N/L in the respective collection timings; SIRI (Systemic immune inflammatory index) was defined as N × (M/L) in the respective collection timings; SII (systemic immune‐inflammatory index) was defined as P × [N/L] in the respective collection timings; finally MLR (Monocyte to Lymphocyte ratio) was defined as M/L in the respective collection timings.

### 2.5 Futile Recanalization and Effective Recanalization

We defined futile recanalization as LVO patients experiencing a 90‐day poor outcome (mRS 3–6) despite successful recanalization (mTICI ≥ 2b) after MT and effective recanalization as LVO patients achieving a 90‐day good outcome (mRS ≤ 2) with successful recanalization after MT.

### 2.6 Statistical Analysis

Categorical variables are presented as count and percentage, continuous variables as mean and standard deviation, or median and interquartile range according to normal distribution. We used inverse probability weighting (IPW) to balance the baseline characteristics of the exposed and unexposed cohorts (FR vs. ER groups), aiming to reduce the confounding factors on study outcomes. To reduce confounding and retain the entire study population, we applied IPW rather than propensity score matching, which would have led to the exclusion of a substantial proportion of unmatched patients. IPW allows the creation of a pseudo‐population in which baseline covariates are balanced across outcome groups without loss of sample size. Covariates included in the weighting model were selected a priori based on clinical relevance and existing literature and comprised age, sex, hypertension, diabetes, hypercholesterolemia, smoking, known atrial fibrillation, NIHSS at admission, ASPECTS, occlusion site, anesthesia type, onset‐to‐recanalization time, bridging therapy, first‐pass success, and post‐procedural hemorrhagic transformation. A detailed methodological explanation of the IPW estimation process is available in the Supplemental Methods. In brief, weights were obtained by calculating the probability of being in the group of FR vs. ER while controlling for a set of relevant variables that could have influenced the outcome. The weights obtained were then used to balance the baseline covariates, therefore creating a pseudo‐population independent of the measured confounders (i.e., pseudo‐randomization) [32]. Statistical comparisons were performed in the weighted population between patient groups using the *χ* ^2^ test, Fisher's exact test, Student's t‐test, and Mann–Whitney *U* as indicated for dichotomous or continuous variables. In order to identify stable predictors in multivariate models, we adopted the least absolute shrinkage and selection operator (LASSO) regression. LASSO linear/gaussian regression allowed the selection of measured biomarkers that are most associated with FR stratified by age groups (< 80 and ≥ 80 years) in the weighted population. Further, we conducted a crude logistic regression analysis to examine the unadjusted association of those markers with FR. Variables with a significant association with this study outcome (*p* ≤ 0.05) were considered for multivariate logistic regression analysis, with statistical significance set at *p* < 0.05. Adjusted odds ratios (ORs) with 95% confidence intervals (CIs) were obtained. OR regression with restricted cubic splines was employed to model the continuous relationship between measured biomarkers of interest and the OR of FR. Knots for the splines were set at the 10th, 50th, and 90th percentiles of the biomarker of interest. The median value of the biomarker was used as the reference, and ORs for varying levels were estimated relative to this reference. Log ORs were computed and exponentiated to yield ORs, with 95% confidence intervals (CIs) calculated using the standard errors of the log ORs. CIs were derived by adjusting each log OR by ± 1.96 times the standard error, then exponentiating the bounds. To identify a clinical cut‐off for the measured biomarkers of interest, the OR curve was examined to locate values where the OR approximated 1, indicating no significant increase or decrease in risk. The measured biomarker level closest to OR = 1 was selected as a potential cut‐off point for stratification. To assess whether clinical variables modify the effect of inflammatory biomarkers on the outcome of futile recanalization, we performed an interaction analysis on the IPW cohort. The analysis was stratified by age (< 80 years vs. ≥ 80 years).

For each age group, we tested interaction terms between inflammatory biomarkers and the pre‐specified clinical variables: administration of thrombolysis, hypertension, diabetes mellitus, and time to recanalization (dichotomized based on the median value).

For each model, we estimated the odds ratio (OR) for the interaction term with corresponding 95% confidence intervals (CIs) and interaction *p*‐values. Statistical significance was set at a *p*‐value of < 0.05. R Studio (version 2024.4) was used for statistical analysis.

## 3 Results

**FIGURE 1:**
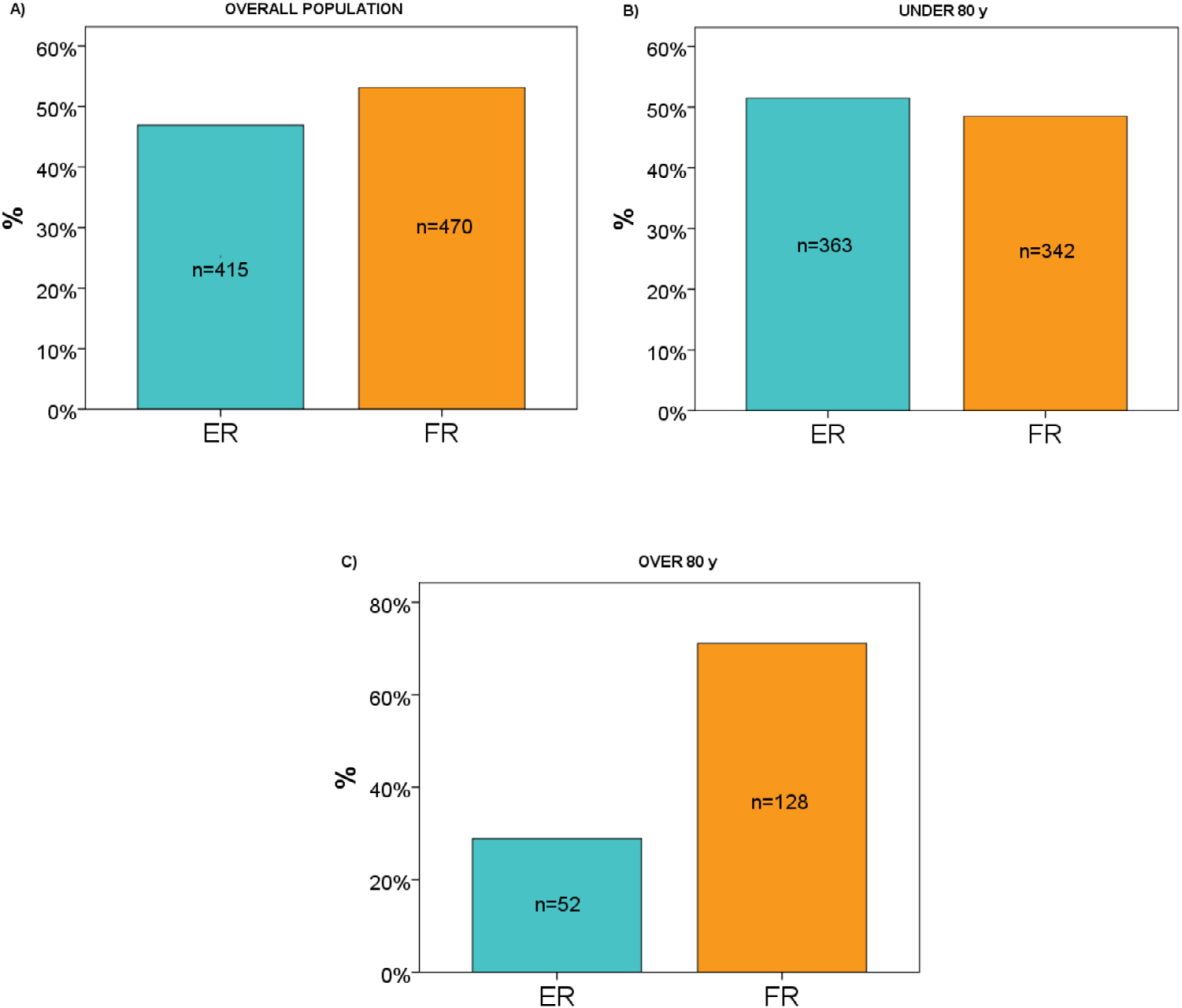
Futile recanalization (FR) and Effective recanalization (ER) rate in the overall population (A), in patients aged below 80 years (B), and in patients over 80 years (C).

Overall, 885 patients with acute ischemic stroke due to LVO achieved successful recanalization after MT and were included in the analysis, of whom 415 (47%) experienced effective recanalization (EF) and 470 (53%) experienced futile recanalization (FR) (Figure S1; Figure 1). As reported in Figure 1, 342 (49%) of patients aged below 80 years experienced FR, while 128 (71%) of patients ≥ 80 years had FR after MT. Table 1 reports unweighted and weighted results for baseline, clinical, and treatment characteristics. Overall, good balance was obtained for all major baseline variables of interest (Figure S2; Table 1). The 90‐day death rate in the overall population was 15% (*n* = 130). The main frailty score assessed with the CSF scale for patients ≥ 80 years was 1.90 (0.73).

**TABLE 1:** Baseline characteristics.

	Overall population (*N* = 885)	FR (*N* = 470)	ER (*N* = 415)	SMD unweighted	SMD weighted
Demographics					
Age, years [median (IQR)]	69 (56–78)	72 (60–80)	65 (54–74)	0.379	−0.033
Female sex [*n*, (%)]	411 (46)	223 (47)	188 (45)	0.021	0.001
Hypertension [*n*, (%)]	483 (55)	289 (62)	194 (47)	0.147	0.013
Diabetes mellitus [*n*, (%)]	176 (20)	109 (23)	67 (16)	0.070	0.001
Hypercholesterolemia [*n*, (%)]	306 (35)	176 (37)	130 (31)	0.061	0.001
Known Atrial fibrillation [*n*, (%)]	199 (23)	122 (26)	77 (19)	0.074	0.011
AFDAS [*n*, (%)]	163 (18)	91 (19)	72 (17)	0.020	−0.002
Coronary artery disease [*n*, (%)]	140 (16)	83 (17)	57 (14)	0.039	0.002
Congestive heart failure [*n*, (%)]	73 (8)	43 (9)	30 (7)	0.019	−0.001
Current smoking [*n*, (%)]	145 (16)	59 (13)	86 (20)	−0.081	0.002
ETOH [*n*, (%)]	118 (13)	60 (13)	58 (14)	−0.012	−0.007
Previous TIA/ischemic stroke [*n*, (%)]	146 (17)	90 (19)	56 (14)	−0.001	−0.001
Previous ICH [*n*, (%)]	6 (1)	3 (1)	3 (1)	−0.001	−0.001
Admission therapy					
Anticoagulation on admission [*n*, (%)]	114 (13)	63 (13)	51 (12)	0.011	0.013
Antiplatelet therapy on admission [*n*, (%)]	175 (20)	110 (23)	65 (16)	0.077	−0.001
Stroke characteristics					
Known onset [*n*, (%)]	741 (84)	383 (82)	358 (86)	−0.047	0.014
Pre stroke care [*n*, (%)]				0.024	0.003
Mothership	347 (39)	179 (38)	168 (41)		
Drip‐and‐ship	538 (61)	291 (62)	247 (59)		
NIHSS on admission [median (IQR)]	17 (12–21)	18 (13–22)	16 (10–20)	0.390	0.008
Site of occlusion [*n*, (%)]					
Distal ICA	91 (10)	47 (10)	44 (11)		
M1	558 (63)	300 (64)	258 (62)		
M2	131 (15)	62 (13)	69 (17)		
Tandem occlusion	105 (12)	61 (13)	44 (11)		
Proximal (Distal ICA, M1, Tandem) vs. distal (M2)	754 (85) vs. 131 (15)	408 (87) vs. 62 (13)	346 (83) vs. 69 (17)	−0.034	0.005
Onset‐to‐door	216 (94–282)	219 (100–285)	212 (87–275)	0.031	0.059
Onset‐to‐groin	255 (205–320)	271 (210–331)	245 (195–310)	0.100	0.015
Onset to recanalization	287 (142–371)	309 (125–380)	275 (142–362)	0.049	0.016
First pass technique used, *n* (%)				−0.036	−0.001
Aspiration	646	354	292		
Stent retriever	239	116	123		
*N* of passes [median (IQR)]	1 (1–2)	1 (1–2)	1 (1–2)	0.008	−0.014
First pass successful [*n*, (%)] (*N* = 302)	169 (56)	86 (56)	83 (56)	0.024	−0.019
General anesthesia [*n*, (%)]	698 (79)	393 (84)	305 (74)	0.101	−0.004
Bridging therapy [*n*, (%)]	690 (67)	301 (64)	289 (70)	−0.055	−0.007
ASPECTS [median (IQR)]	9 (8–10)	9 (7–10)	9 (8–10)	−0.360	0.019
Post MT Hemorrhagic transformation [*n*, (%)]	235 (27)	165 (35)	70 (17)	0.182	−0.005
sICH [*n*, (%)]	42 (5)	36 (7)	6 (1)	0.062	−0.001

Abbreviations: AFDAS, atrial fibrillation detected after stroke; ETOH, ethanol abuse; ICH, intra cerebral hemorrhage; NIHSS, National Institutes of Health Stroke Scale; sICH, symptomatic intracerebral hemorrhage; TIA, transient ischemic attack; TICI, modified thrombolysis in cerebral infarction classification.

Table 2 shows the comparison of the blood test results between FR and ER in the weighted population performed on admission and at 24 h from the index event. Patients with FR statistically differed in terms ofHb (*p* = 0.003), Neutrophil count (*p* = 0.011), Lymphocyte count (*p* < 0.001), Eosinophil count (*p* = 0.045), CRP (*p* = 0.013), Glycemia (*p* < 0.001), HbA1c (*p* < 0.001), NLR (*p* < 0.001), SIRI (*p* < 0.001), SII (*p* < 0.001) and MLR (*p* < 0.001) on admission compared to ER. At 24‐h, the two groups differed significantly in terms of WBC(*p* < 0.001), RBC (*p* = 0.037), Hb (*p* = 0.003), Neutrophil count(*p* < 0.001), Lymphocyte count (*p* < 0.001), Eosinophil count (*p* < 0.001), CRP (*p* < 0.001), NLR (*p* < 0.001), SIRI (*p* < 0.001), SII (*p* < 0.001) and MLR (*p* < 0.001).

**TABLE 2:** Blood test in the weighted population.

	FR (*N* = 470)	ER (*N* = 415)	*p*
Admission test [median (IQR)]			
WBC (×10^9/L^)	8.75 (3.87)	8.50 (3.35)	0.112
RBC (×10^12/L^)	4.40 (0.81)	4.47 (0.67)	0.056
Hb (g/L)	134 (22)	137 (20)	**0.003**
PLT (×10^9/L^)	212 (75)	216 (85)	0.414
Neutrophils count (×10^9/L^)	6.52 (4.05)	5.80 (4.05)	**0.011**
Lymphocytes count (×10^9/L^)	1.28 (1.09)	1.50 (1.20)	**< 0.001**
Monocytes count (×10^9/L^)	0.60 (0.38)	0.56 (0.30)	0.663
Eosinophils count (×10^9/L^)	0 (0.12)	0.10 (0.13)	**0.045**
ESR (mm/h)	10 (20)	9 (13)	0.091
CRP (mg/L)	3.1 (5.37)	2.74 (5.01)	**0.013**
Glycemia (mmol/L)	7.2 (2.77)	6.38 (1.80)	**< 0.001**
HbA1c (mmol/mol)	40 (9)	39 (7)	**< 0.001**
NLR	4.64 (5.97)	3.72 (4.75)	**< 0.001**
SIRI	2.64 (3.54)	2.08 (2.62)	**< 0.001**
SII	1110.33 (1344.47)	817.33 (1224.32)	**< 0.001**
MLR	0.42 (0.31)	0.35 (0.25)	**< 0.001**
24 h test [median (IQR)]			
WBC (×10^9/L^)	9.75 (3.86)	8.59 (3.46)	**< 0.001**
RBC (×10^12/L^)	4.09 (0.79)	4.16 (0.67)	**0.037**
Hb (g/L)	123 (24.5)	127.0 (21.8)	**0.003**
PLT (×10^9/L^)	193 (70)	200 (75)	0.154
Neutrophils count (×10^9/L^)	7.66 (3.93)	6.2 (3.4)	**< 0.001**
Lymphocytes count (×10^9/L^)	1.20 (0.70)	1.49 (0.72)	**< 0.001**
Monocytes count (×10^9/L^)	0.70 (0.40)	0.70 (0.39)	0.103
Eosinophils count (×10^9/L^)	0 (0.08)	0.03 (0.10)	**< 0.001**
CRP (mg/L)	9.58 (18.21)	6.47 (10.90)	**< 0.001**
NLR	5.83 (5.16)	4.14 (2.89)	**< 0.001**
SIRI	4.13 (4.64)	2.76 (2.88)	**< 0.001**
SII	1243.74 (1266.87)	889.79 (853.91)	**< 0.001**
MLR	0.55 (0.42)	0.44 (0.32)	**< 0.001**

*Note:* Bold values are statistically significant (*p* < 0.05).

Abbreviations: CRP, C reactive protein; ESR, erythrocyte sedimentation rate; Hb, hemoglobin; HbA1c, glycated hemoglobin; LR, neutrophil‐to‐lymphocyte ratio; PLT, platelets; RBC, red blood count; WBC, white blood count.

### 3.1 LASSO Regression Analysis to Identify Inflammatory Predictors of Futile Recanalization

**FIGURE 2:**
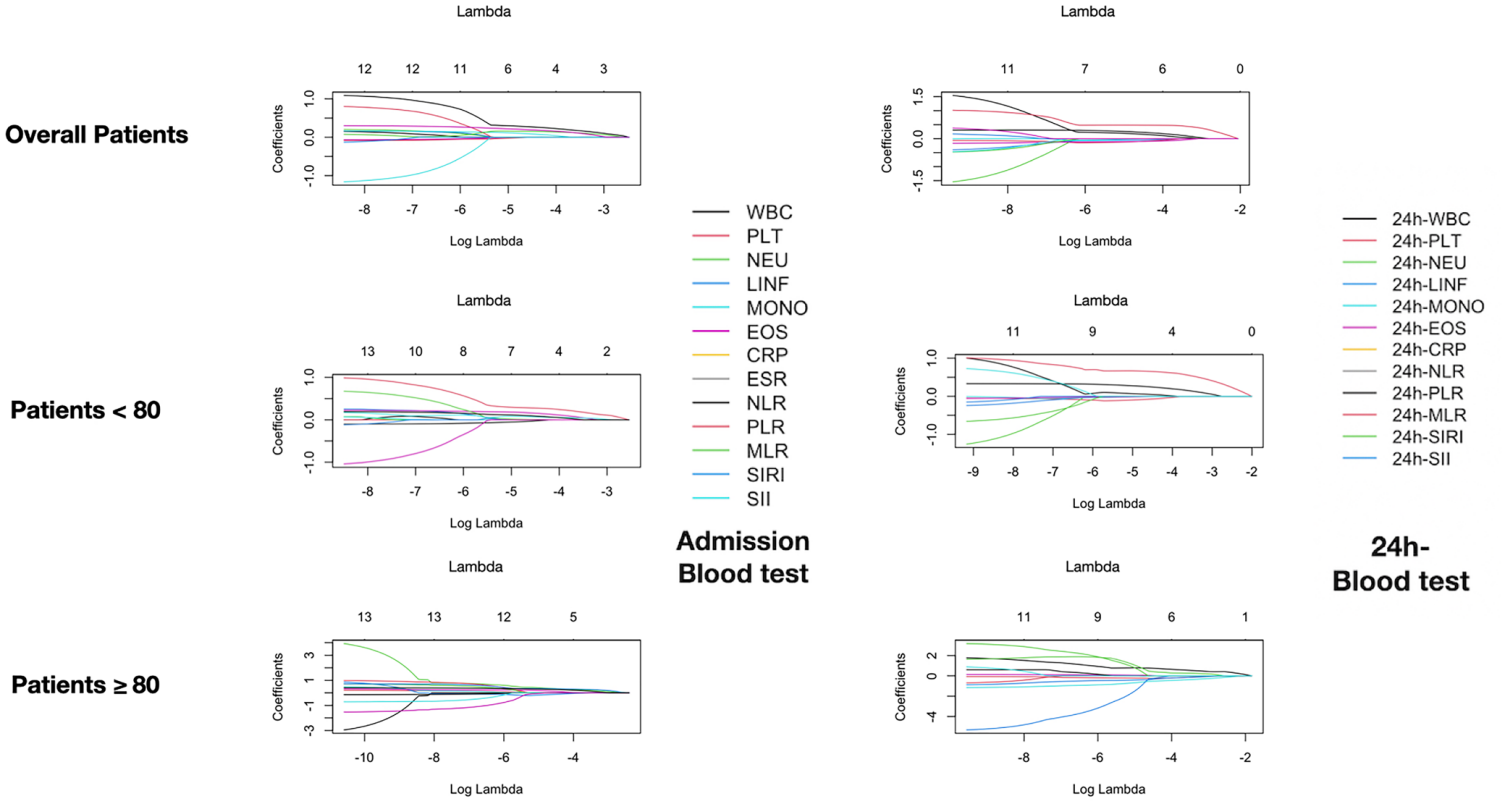
LASSO linear regression models for futile recanalization in the overall patients and by age subgroups. Panels show the coefficients of the LASSO linear regression analysis for the prediction of futile recanalization in the overall patients and by age subgroups at increasing lambda values. The regularization parameter lambda indicates the selection power of each model, i.e., the higher the lambda values, the lower the number of variables of the model. Lambda values at 1 standard error from the optimal one, selected by cross‐validation, are displayed as red dashed lines.

We conducted a LASSO regression analysis to identify the variables most strongly associated with futile recanalization, stratified by age groups (< 80 and ≥ 80 years) in the weighted population. The values of the LASSO coefficients for each of the included variables after variation of the regularization parameter lambda are shown in Figure 2. The plot shows how the relative importance of each variable changes at increasing model size. At optimal model size (best lambda value determined by cross‐validation + 1 standard error), the variables that were most associated with futile recanalization in the whole weighted population were admission eosinophil count, NLR, MLR and ESR, and 24‐h WBC, PLT, neutrophil count, lymphocyte count, monocyte count, eosinophil count, CRP, and NLR. In the subgroup of patients with age < 80 years, these were admission eosinophil count, NLR, and SIRI, as well as 24‐h WBC, PLT, lymphocyte count, monocyte count, eosinophil count, CLR, and NLR. In patients aged ≥ 80 years, the variables were admission SIRI and 24‐h WBC, PLT, neutrophil count, lymphocyte count, eosinophil count, CRP, MLR, and SIRI. The coefficient estimates relative to the optimal model size are reported in Table S1.

### 3.2 Inflammatory Predictors of Futile Recanalization in the Whole Weighted Population

**FIGURE 3:**
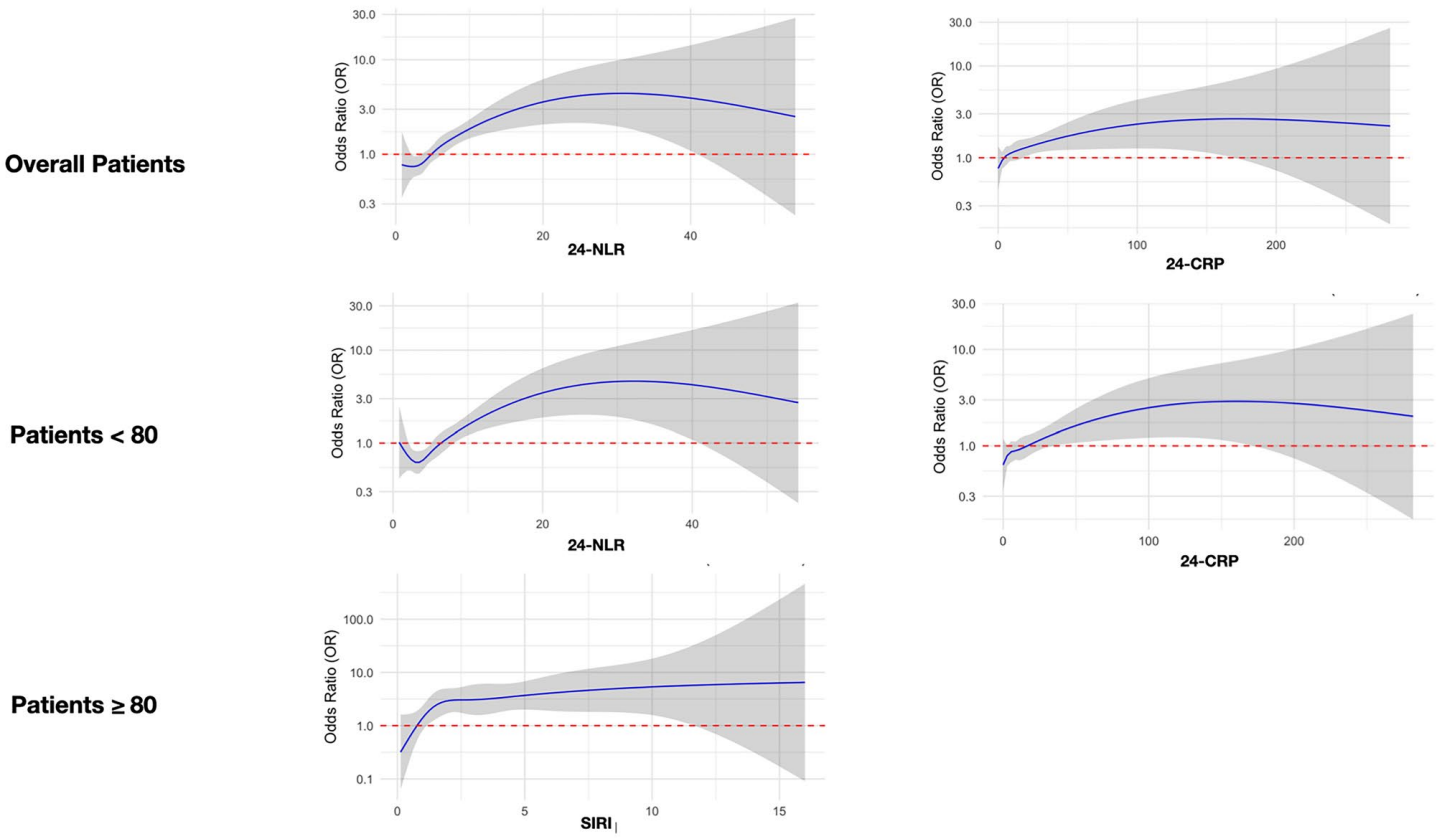
The restricted cubic spline curve of 24h‐NLR, 24‐CRP, and SORI in acute LVO patients receiving MT for predicting futile recanalization. Blue solid line indicates the odd ratio, and gray lines indicate the 95% confidence interval bands. The reference point is determined with knots placed for the splines at the 10th, 50th, and 90th percentiles of the biomarker of interest.

Table 3 shows the logistic univariate and multivariate regression analysis to determine the association between admission and 24‐h inflammation markers and FR in the whole weighted population. Among the inflammatory markers on admission, we did not find any significantly associated with the risk of FR. Among the inflammatory markers at 24‐h, 24‐h CRP (OR 1.01, 95% CI 1.01–1.02, *p* = 0.018) and 24‐h NLR (OR 1.11, 95% CI 1.02–1.22, *p* = 0.019) were found to be independent predictors of FR after multivariate analysis. The logistic regression analysis with restricted cubic splines revealed a non‐linear association between 24‐h CRP and 24‐h NLR and the OR of FR At the point where the OR approximated 1, the analysis identified a cut‐off of 8.55 for 24‐h CRP (*p* non linearity = 0.033) and 4.58 for 24‐h NLR (*p* non linearity = 0.043) (Figure 3).

**TABLE 3:** Multivariate binary logistic regression for FR in the weighted population (all ages included) and in age subgroups (< 80 y and ≥ 80 y).

Overall population						
	Univariate			Multivariate		
	OR	95% CI	*p*	OR	95% CI	*p*
Admission test						
Eosinophils	0.51	0.17–1.15	0.193			
NLR	1.07	1.03–1.10	**< 0.001**	1.05	0.99–1.09	0.073
MLR	2.89	1.61–5.37	**< 0.001**	2.04	0.75–5.73	0.165
ESR	1.01	0.99–1.02	0.083			
24 h test						
WBC	1.21	1.10–1.35	**< 0.001**	1.39	0.95–2.47	0.179
PLT	0.99	0.99–1.00	0.720			
Neutrophils	1.24	1.12–1.38	**< 0.001**	0.740	0.39–1.15	0.271
Lymphocyte	0.44	0.27–0.66	**< 0.001**	0.720	0.36–1.20	0.296
Monocyte	0.93	0.68–1.19	0.545			
Eosinophils	0.04	0.01–0.28	**< 0.001**	0.232	0.02–1.82	0.192
CRP	1.01	1.00–1.02	**0.003**	1.01	1.01–1.02	**0.018**
NLR	1.14	1.09–1.19	**< 0.001**	1.11	1.02–1.22	**0.019**

Under 80 y						
	Univariate			Multivariate		
	OR	95% CI	*p*	OR	95% CI	*p*
Admission test						
Eosinophils	0.69	0.24–1.43	0.404			
NLR	1.06	1.03–1.10	**< 0.001**	1.03	0.97–1.09	0.258
SIRI	1.10	1.05–1.18	**< 0.001**	1.07	0.98–1.17	0.145
24 h test						
WBC	1.14	1.08–1.21	**< 0.001**	1.04	0.94–1.15	0.480
PLT	1.00	0.99–1.00	0.766			
Lymphocyte	0.54	0.40–0.71	**< 0.001**	0.92	0.54–1.51	0.757
Monocyte	0.98	0.70–1.36	0.904			
Eosinopils	0.08	0.01–0.50	**0.011**	0.67	0.06–5.87	0.721
CRP	1.01	1.01–1.03	**0.004**	1.01	1.01–1.02	**0.018**
NLR	1.14	1.09–1.20	**< 0.001**	1.11	1.03–1.22	**0.013**

Over 80 y						
	Univariate			Multivariate		
	OR	95% CI	*p*	OR	95% CI	*p*
Admission test						
SIRI^a^	1.24	1.06–1.50	**0.015**			
24 h test						
WBC^a^	1.46	1.18–1.85	**< 0.001**	1.11	0.64–2.08	0.705
PLT^a^	0.99	0.99–1.00	0.353			
Neutrophils^a^	1.49	1.18–1.97	**0.002**	1.40	0.71–2.65	0.313
Lymphocyte^a^	0.66	0.40–1.00	0.075			
Eosinophils^a^	0.01	0.00–0.12	**< 0.001**	0.01	0.01–0.39	0.071
CRP^a^	1.00	0.98–1.02	0.732			
MLR^a^	2.58	0.67–12.90	0.206			
SIRI^a^	1.18	0.99–1.44	0.067			

*Note:* Bold values are statistically significant (*p* < 0.05).

^a^ Adjusted for clinical frailty scale (CSF).

### 3.3 Inflammatory Predictors of Futile Recanalization in Patients Aged < 80 Years

Subgroup analysis by age demonstrated that patients aged < 80 years, 24‐h CRP (OR 1.01, 95% CI 1.01–1.02, *p* = 0.018) and 24‐h NLR (OR 1.11, 95% CI 1.03–1.22, *p* = 0.013) were significantly associated with the risk of FR after multivariate analysis (Table 3). The logistic regression analysis with restricted cubic splines revealed a non‐linear association between 24‐h CRP and 24‐h NLR and the OR of FR in the subgroup of patients aged < 80 years. At the point where the OR approximated 1, the analysis identified a cut‐off of 17.09 (*p* non linearity = 0.039) for 24‐h CRP and 5.59 for 24‐h NLR (*p* non linearity = 0.008) (Figure 3).

### 3.4 Inflammatory Predictors of Futile Recanalization in Patients Aged ≥ 80 Years

Subgroup analysis by age demonstrated that in patients aged *≥* 80 years, admission SIRI was significantly associated with the risk of FR (OR 1.24, 95% CI 1.06–1.50, *p* = 0.015) (Table 3). The logistic regression analysis with restricted cubic splines revealed a non‐linear association between admission SIRI and the OR of FR in the subgroup of patients aged *≥* 80 years. At the point where the OR approximated 1, the analysis identified a cut‐off of 2.53 for admission SIRI (*p* non‐linearity = 0.006) (Figure 3). This analysis was adjusted for the level of CSF.

### 3.5 Interaction Analysis Between Inflammatory Predictors and Clinical Variables

Our analysis showed that in patients younger than 80 years, a statistically significant interaction was observed between CRP at 24 h and use of thrombolytic (OR 0.97 (95% CI: 0.95–1.00, *p* = 0.0359)), suggesting that the association between CRP24 levels and 90‐day outcomes may differ based on thrombolysis status. No other interactions in this age group reached statistical significance (Tables S2 and S3).

## 4 Discussion

Our analysis revealed distinct age‐specific inflammatory responses that are associated with the likelihood of achieving a favorable outcome despite successful recanalization after MT for LVO ischemic stroke. One of the key findings of our study is that certain biomarkers, particularly CRP and NLR measured at 24 h post‐MT, were significantly associated with FR across all age groups. However, further subgroup analyses delineated differences between younger patients (< 80 years) and the elderly (≥ 80 years) in terms of inflammatory predictors of FR. While 24‐h CRP and NLR emerged as predictors in the patients below 80, admission SIRI resulted in being a significant predictor of FR, specifically among octogenarian patients. These findings underscore the nuanced interplay between age and inflammatory responses in determining the risk of FR after MT.

To date, there is limited evidence regarding the role of systemic inflammatory biomarkers in predicting FR [10, 11, 12, 13, 14, 15, 16, 17]. A previous single‐centre study on 324 patients [17] investigated the role of neutrophils in the prediction of FR after MT. They found an association between admission neutrophil count and FR while they excluded NLR from the multivariate analysis. Conversely, we included NLR and other indices, on admission and at 24 h, in our study analysis. This was based on the fact that NLR proved to be more effective than individual neutrophil or lymphocyte counts for outcome prediction, as it reflects the dynamic interplay between these two key immune parameters [33]. Another study [16] investigated the contribution of neutrophil and lymphocyte counts to hemorrhagic complications and functional outcome in stroke patients treated with MT with varying degrees of collateral circulation and reperfusion. The authors concluded that the degree of reperfusion was associated with increased neutrophil counts and NLR. In contrast to this analysis, we focused primarily on the association between inflammatory biomarkers and the risk of FR. Moreover, it is noteworthy to mention that most of the previous studies did not account for conditions that could influence inflammatory markers, such as infections or the use of immunosuppressive medications, within their exclusion criteria, and often relied on relatively small single‐centre cohorts of patients. We also used an IPW analysis to balance the baseline characteristics of the two groups (ER vs. FR), aiming to reduce the confounding effect of a potential bias by indication, given the retrospective and non‐randomized nature of the study.

An original of our analysis is the significant association between elevated CRP levels and an increased risk of FR in patients under 80 years of age. Conversely, we demonstrated that SIRI serves as an independent predictor of FR specifically in patients over 80, with no significant association observed in younger patients. Recently, CRP has been proven to be an independent predictor of FR in patients undergoing recanalization therapies (MT and/or IVT) [34]. Similarly, it has been demonstrated that elevated CRP levels prior to MT were significantly associated with worse outcomes and higher mortality in stroke patients [35]. The relationship between CRP and stroke outcomes can be attributed to a two‐step mechanism. Initially, tissue damage releases local inflammatory triggers, activating thrombo‐inflammatory pathways [36]. Subsequently, CRP directly activates the complement system, as shown in preclinical studies [37]. This amplification of the inflammatory response exacerbates tissue injury, ultimately contributing to poorer outcomes.

The SIRI biomarker, which combines neutrophil, monocyte, and lymphocyte counts, effectively captures the dynamic interplay between critical components of the immune system. To date, only one study has investigated the role of SIRI in predicting FR post MT [11], focusing solely on admission blood samples. In a single‐centre analysis of 184 patients treated with MT, it was documented that higher admission SIRI increased the risk of poor outcome at 3 months despite complete or near‐complete recanalization [11]. In contrast to the authors, we estimated SIRI through peripheral blood cell counts obtained at two time points rather than on a single measurement with the aim of exploring the dynamic evolution of the inflammatory marker. Based on our findings, we could not clarify the mechanism of higher SIRI detection in elderly patients with FR. Nevertheless, SIRI elevation in elderly patients may reflect immune aging, neurodegeneration, and hyper‐acute stroke responses. Lymphocyte reduction in older adults is documented [38], and neurodegenerative conditions like Alzheimer's disease (AD) provide insights. A study [39] showed monocyte infiltration into AD brains, likely due to blood–brain barrier dysfunction, with similar findings in hypertensive, aged rats [40]. SIRI is strongly associated with cognitive decline in older adults [41], and another study [42] found an inverse relationship between monocyte levels and AD. Further research is needed to explore the link between SIRI and FR in elderly patients. In the elderly subgroup, we retrospectively assessed pre‐stroke frailty using the CSF to account for baseline vulnerability that may influence both functional recovery and systemic inflammation. The inclusion of CSF in the multivariate model did not substantially alter the results, confirming that admission SIRI remains a significant predictor of futile recanalization in this population. This might support the notion that systemic inflammation and frailty may represent partially independent pathways contributing to poor outcomes after mechanical thrombectomy in older adults.

Finally, our study identified NLR as an independent predictor of FR in patients undergoing MT, regardless of the age of the patients. NLR reflects both innate and adaptive immunity. Neutrophils peak at 24–48 h, contributing to brain damage through reactive oxygen species and pro‐inflammatory cytokines, while T‐lymphocytes play a key role in modulating inflammation and tissue repair. While previous studies [11, 12, 13, 14, 15, 17] focused solely on admission or early (within 24 h) NLR, our study evaluated the dynamic changes of NLR in FR. The association between NLR and FR may be attributed to neutrophil infiltration in small distal vessels, leading to stalled circulation and the so‐called no‐reflow phenomenon [43]. Another contributing mechanism might involve neutrophil extracellular traps (NETs). It has been demonstrated [44] that there is a correlation between NLR and the presence of NETs within thrombi retrieved during MT. Based on this evidence, we hypothesize that NLR may not only reflect thrombus composition but also influence NET production in the distal microcirculation. This could explain why, despite successful recanalization of proximal large vessels, blood flow fails to adequately perfuse the ischemic tissue. Indeed, as suggested before [44], novel therapies such as DNase‐1, which disrupt NETs, may improve microcirculatory reperfusion and enhance clinical outcomes. Further research is necessary to validate our findings and explore the therapeutic potential of NET‐disrupting agents in the context of hyperacute stroke treatment.

Our analysis has several strengths: (1) a large patient cohort, (2) a multicentre study design, (3) stringent exclusion criteria ensuring robust patient selection, and (4) the clinical relevance and applicability of the findings. However, there are notable limitations. The retrospective design and reliance on routine clinical data may introduce biases and limit generalizability. Despite efforts to address confounding through inverse probability weighting, residual confounding cannot be entirely ruled out. Biomarker assessments were limited to admission and 24‐h time points. While these reflect early inflammatory changes relevant to acute management, they do not capture the full temporal dynamics of the inflammatory response. This limitation is partly due to the fact that 61% of patients in our cohort were treated under a drip‐and‐ship model and were transferred back to their referring centers shortly after thrombectomy, making standardized follow‐up blood sampling at 48–72 h unfeasible. Future prospective studies with extended biomarker sampling are needed to better characterize late‐phase inflammatory trajectories and their clinical implications. Another limitation is the lack of a standardized operating procedure (SOP) across participating centers. While all centers involved are high‐volume stroke units adhering to international guidelines, variability in thrombectomy techniques, peri‐procedural management, post‐stroke care, and laboratory procedures may have introduced center‐related heterogeneity. Although we attempted to mitigate this through strict inclusion criteria, harmonized data collection, and inverse probability weighting, residual variability across sites cannot be completely excluded. Another limitation of our study is the lack of data on collateral circulation, stroke etiology, and post‐thrombectomy re‐occlusion. Collateral status is a known determinant of tissue survival and has been independently associated with functional outcomes following MT. Its absence in our dataset may have confounded the association between inflammatory biomarkers and futile recanalization, particularly in patients with poor collaterals who are more likely to have extensive ischemic injury regardless of reperfusion success. Moreover, stroke etiology was not systematically captured across centers; different etiologic subtypes may present distinct inflammatory profiles, which could influence both biomarker levels and outcome trajectories. Additionally, we did not collect data on vessel re‐occlusion following MT, which, although infrequent, may contribute to poor outcomes despite initial recanalization and may partially explain some cases classified as futile recanalization. Future studies should incorporate these variables to refine risk stratification models and improve mechanistic understanding.

## 5 Conclusion

Our multicentre study highlights the critical role of systemic inflammatory biomarkers, particularly NLR, CRP, and SIRI, in predicting FR following mechanical thrombectomy. By assessing both admission and 24‐h blood samples, we provide novel insights into the dynamic inflammatory processes contributing to FR and their age‐specific variations. Indeed, we demonstrated that age (using 80 years as a cut‐off) significantly influences biomarker profiles associated with FR. This finding underscores the potential for tailoring stroke management strategies to different age groups, paving the way for personalized medicine approaches. Such insights could also guide the design of future randomized controlled trials aimed at improving outcomes in diverse patient populations. Future research should focus on validating these biomarkers in prospective cohorts and exploring therapeutic interventions, such as targeting neutrophil extracellular traps, to enhance microvascular reperfusion and clinical outcomes in patients undergoing mechanical thrombectomy.

## Author Contributions

**Gabriele Prandin:** conceptualization, writing – original draft, formal analysis, investigation. **Mariarosaria Valente:** writing – review and editing, visualization. **Liqun Zhang:** visualization, writing – review and editing. **Paresh Malhotra:** writing – review and editing, visualization. **Simona Sacco:** writing – review and editing, visualization. **Matteo Foschi:** methodology, writing – review and editing, supervision. **Raffaele Ornello:** methodology, writing – review and editing, visualization. **Edoardo Pirera:** writing – review and editing, methodology. **Francesco Toraldo:** investigation, writing – review and editing. **Domenico Maisano:** investigation, writing – review and editing. **Caterina Del Regno:** investigation, writing – review and editing. **Filippo Komauli:** investigation, writing – review and editing. **Adelaida Gartner Jaramillo:** investigation, writing – review and editing. **Hakam AL‐Karadsheh:** investigation, writing – review and editing. **Hamza Zahid:** investigation, writing – review and editing. **Piers Klein:** investigation, writing – review and editing. **Mohamad Abdalkader:** investigation, writing – review and editing. **Paolo Manganotti:** writing – review and editing, visualization, supervision. **Kyriakos Lobotesis:** writing – review and editing. **Thanh N. Nguyen:** writing – review and editing, visualization. **Soma Banerjee:** writing – review and editing, project administration, visualization. **Gian Luigi Gigli:** writing – review and editing, visualization. **Giovanni Merlino:** writing – review and editing, methodology, validation. **Lucio D'Anna:** conceptualization, investigation, writing – review and editing, methodology, formal analysis, project administration, supervision, data curation.

## Conflicts of Interest

The authors declare no conflicts of interest.

## Supporting information


Data S1.

## Data Availability

The data that support the findings of this study are available from the corresponding author upon reasonable request.
