# Supplementary material for: Age‐Specific Differences in Inflammatory Biomarkers and Their Impact on Futile Recanalization After Mechanical Thrombectomy: An Inverse Probability Weighting Analysis
Source: Eur J Neurol. 2025 May 12;32(5):e70182. doi: 10.1111/ene.70182 (PMC12067390; doi:10.1111/ene.70182)

**SUPPLEMENTARY MATERIAL**

**Table S1.** Coefficient estimates of least absolute shrinkage and selection operator (LASSO) regression models.

| **Study Group** | **Penalty tuning parameter** | **Stable predictors** | **Coefficient estimates** |
| --- | --- | --- | --- |
| **Overall Patients** | **Optimal lambda** | Eosinophils count  NLR  MLR  ESR | 0.05510507  0.24028234  0.14268190  0.16805211 |
|  |  | 24h-WBC  24h-PLT  24h- Lymphocytes count  24h- Eosinophils count  24h-CRP  24h-NLR | 0.10890997  -0.05689008  -0.02183518  -0.08186243  0.18024246  0.47874338 |
| **Patients < 80 years** | **Optimal lambda** | Eosinophils count  NLR  SIRI  ESR | 0.11724977  0.24672102  0.05757686  0.05513504 |
|  |  | 24h- CRP  24h-NLR | 0.16063567  0.56829695 |
| **Patients ≥80 years** | **Optimal lambda** | SIRI | 0.000000001 |
|  |  | 24h-WBC  24h-PLT  24h-Neutrophils count  24h-Lymphocytes count  24h- Eosinophils count  24h- CRP  24h-MLR  24h-SIRI | 0.74870796  -0.20827788  1.61685452  -0.43483517  -0.80122827  0.08027788  1.37922465  -2.37790773 |

Data represent coefficient estimates of LASSO linear regression models by selecting the models with optimal lambda values. Two models were built by accounting as response futile recanalization and as predictors the variables White Blood Cell count (WBC), C-reactive protein (CRP), neutrophil-to-lymphocyte ratio (NLR), platelet-to-lymphocyte ratio (PLR), monocyte-to-lymphocyte ratio (MLR), systemic inflammation response index (SIRI), and systemic immune-inflammation index (SII), neutrophil count, platelet count (PLT), lymphocyte count and monocyte, Erythrocyte Sedimentation Rate (ESR), Eosinophils counton admission and at 24 hours after the index event. We reported only stable predictors with coefficient estimates values different from 0stratified by age groups (<80 and ≥80 years) in the weighted population.

**Table S2. Interaction analysis between inflammatory biomarkers and clinical covariates in patients aged below 80 years.**

| **Biomarker** | **Clinical variable** | **OR of interaction** | **CI 95%** | **P value** |
| --- | --- | --- | --- | --- |
| **Admission test** |  |  |  |  |
| Eosinophils | Bridging therapy | 1.14 | 0.13-10.02 | 0.904 |
| Eosinophils | Hypertension | 2.02 | 0.16-25.47 | 0.585 |
| Eosinophils | Diabetes | 0.84 | 0.08-8.86 | 0.881 |
| Eosinophils | Recanalization time | 0.60 | 0.02-19.52 | 0.773 |
| NLR | Bridging therapy | 1.02 | 0.94-1.10 | 0.668 |
| NLR | Hypertension | 1.02 | 0.95-1.09 | 0.593 |
| NLR | Diabetes | 1.12 | 1.00-1.26 | 0.055 |
| NLR | Recanalization time | 1.04 | 0.96-1.14 | 0.325 |
| SIRI | Bridging therapy | 0.99 | 0.87-1.14 | 0.937 |
| SIRI | Hypertension | 1.02 | 0.91-1.15 | 0.761 |
| SIRI | Diabetes | 1.08 | 0.90-1.29 | 0.414 |
| SIRI | Recanalization time | 1.05 | 0.91-1.22 | 0.474 |
| **24h test** |  |  |  |  |
| WBC | Bridging therapy | 1.07 | 0.95-1.20 | 0.282 |
| WBC | Hypertension | 1.08 | 0.96-1.22 | 0.204 |
| WBC | Diabetes | 0.96 | 0.83-1.12 | 0.631 |
| WBC | Recanalization time | 1.00 | 0.86-1.17 | 0.979 |
| PLT | Bridging therapy | 1.00 | 0.99-1.00 | 0.854 |
| PLT | Hypertension | 1.00 | 0.99-1.00 | 0.913 |
| PLT | Diabetes | 1.00 | 0.99-1.00 | 0.210 |
| PLT | Recanalization time | 1.00 | 1.00-1.01 | 0.394 |
| Lymphocyte | Bridging therapy | 0.68 | 0.37-1.23 | 0.200 |
| Lymphocyte | Hypertension | 1.57 | 0.87-2.86 | 0.136 |
| Lymphocyte | Diabetes | 1.37 | 0.73-2.59 | 0.327 |
| Lymphocyte | Recanalization time | 1.10 | 0.55-2.21 | 0.779 |
| Monocyte | Bridging therapy | 0.45 | 0.15-1.35 | 0.156 |
| Monocyte | Hypertension | 0.79 | 0.30-2.06 | 0.630 |
| Monocyte | Diabetes | 1.77 | 0.46-6.82 | 0.406 |
| Monocyte | Recanalization time | 0.93 | 0.19-4.46 | 0.929 |
| Eosinophils | Bridging therapy | 1.33 | 0.02-116.59 | 0.901 |
| Eosinophils | Hypertension | 1.21 | 0.02-79.67 | 0.928 |
| Eosinophils | Diabetes | 37.28 | 0.47-2974.68 | 0.105 |
| Eosinophils | Recanalization time | 1.76 | 0.01-260.79 | 0.825 |
| CRP | Bridging therapy | 0.97 | 0.95-1.00 | **0.036** |
| CRP | Hypertension | 1.02 | 1.00-1.04 | 0.065 |
| CRP | Diabetes | 0.98 | 0.96-1.00 | 0.057 |
| CRP | Recanalization time | 0.98 | 0.95-1.00 | 0.059 |
| NLR | Bridging therapy | 1.08 | 0.99-1.19 | 0.084 |
| NLR | Hypertension | 1.00 | 0.91-1.09 | 0.998 |
| NLR | Diabetes | 0.96 | 0.86-1.06 | 0.379 |
| NLR | Recanalization time | 1.00 | 0.89-1.12 | 0.961 |

**Table S3. Interaction analysis between inflammatory biomarkers and clinical covariates in patients aged 80 years and above.**

| **Biomarker** | **Clinical variable** | **OR of interaction** | **CI 95%** | **P value** |
| --- | --- | --- | --- | --- |
| **Admission test** |  |  |  |  |
| SIRI | Bridging therapy | 0.81 | 0.51-1.27 | 0.351 |
| SIRI | Hypertension | 1.03 | 0.69-1.59 | 0.884 |
| SIRI | Diabetes | 3.26 | 0.88-1.21 | 0.078 |
| SIRI | Recanalization time | 0.86 | 0.57-1.30 | 0.471 |
| **24h test** |  |  |  |  |
| WBC | Bridging therapy | 0.92 | 0.53-1.58 | 0.751 |
| WBC | Hypertension | 0.93 | 0.55-1.56 | 0.774 |
| WBC | Diabetes | 2.78 | 0.56-1.38 | 0.210 |
| WBC | Recanalization time | 1.54 | 0.79-2.99 | 0.204 |
| PLT | Bridging therapy | 1.00 | 0.99-1.00 | 0.554 |
| PLT | Hypertension | 1.01 | 0.99-1.00 | 0.373 |
| PLT | Diabetes | 1.01 | 0.99-1.00 | 0.226 |
| PLT | Recanalization time | 0.98 | 0.97-1.01 | 0.067 |
| Neutrophils | Bridging therapy | 1.31 | 0.78-2.19 | 0.303 |
| Neutrophils | Hypertension | 0.60 | 0.30-1.20 | 0.147 |
| Neutrophils | Diabetes | 3.80 | 0.61-2.35 | 0.151 |
| Neutrophils | Recanalization time | 0.98 | 0.57-1.69 | 0.933 |
| Lymphocyte | Bridging therapy | 1.01 | 0.40-2.57 | 0.985 |
| Lymphocyte | Hypertension | 1.29 | 0.31-5.39 | 0.724 |
| Lymphocyte | Diabetes | 2.26 | 0.16-3.09 | 0.542 |
| Lymphocyte | Recanalization time | 0.77 | 0.29-2.05 | 0.598 |
| Eosinophils | Bridging therapy | 0.01 | 0.00-7.10 | 0.424 |
| Eosinophils | Hypertension | 0.11 | 0.00-6.95 | 0.700 |
| Eosinophils | Diabetes | 79.89 | 0.00-256 | 0.271 |
| Eosinophils | Recanalization time | 0.00 | 0.00-1.17 | 0.248 |
| CRP | Bridging therapy | 0.98 | 0.94-1.02 | 0.311 |
| CRP | Hypertension | 0.97 | 0.95-1.07 | 0.329 |
| CRP | Diabetes | 1.01 | 0.96-1.03 | 0.701 |
| CRP | Recanalization time | 0.99 | 0.96-1.03 | 0.608 |
| MLR | Bridging therapy | 0.38 | 0.01-1.27 | 0.588 |
| MLR | Hypertension | 0.66 | 0.03-1.57 | 0.798 |
| MLR | Diabetes | 85.14 | 0.07-1.04 | 0.220 |
| MLR | Recanalization time | 2.58 | 0.09-7.38 | 0.579 |
| SIRI | Bridging therapy | 0.85 | 0.55-1.32 | 0.473 |
| SIRI | Hypertension | 0.85 | 0.54-1.33 | 0.483 |
| SIRI | Diabetes | 10.31 | 0.56-1.88 | 0.116 |
| SIRI | Recanalization time | 1.08 | 0.71-1.63 | 0.725 |

**Figure S1**. Study algorithm.


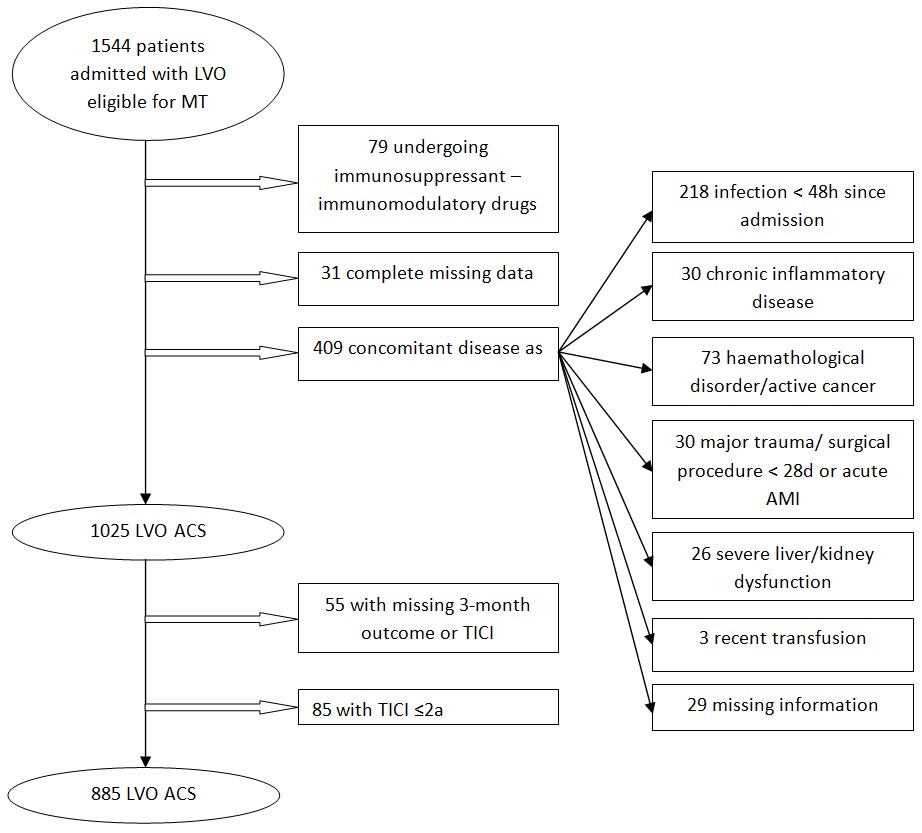


**Legend**: ACS: anterior circulation strokes; LVO: large vessel occlusion; MT: mechanical thrombectomy, AMI: acute myocardial infarction (included ST elevation and non ST elevation myocardial infarction) TICI: modified thrombolysis in cerebral infarction classification

**Figure S2**: Standardized Mean Difference between unweighted and weighted population


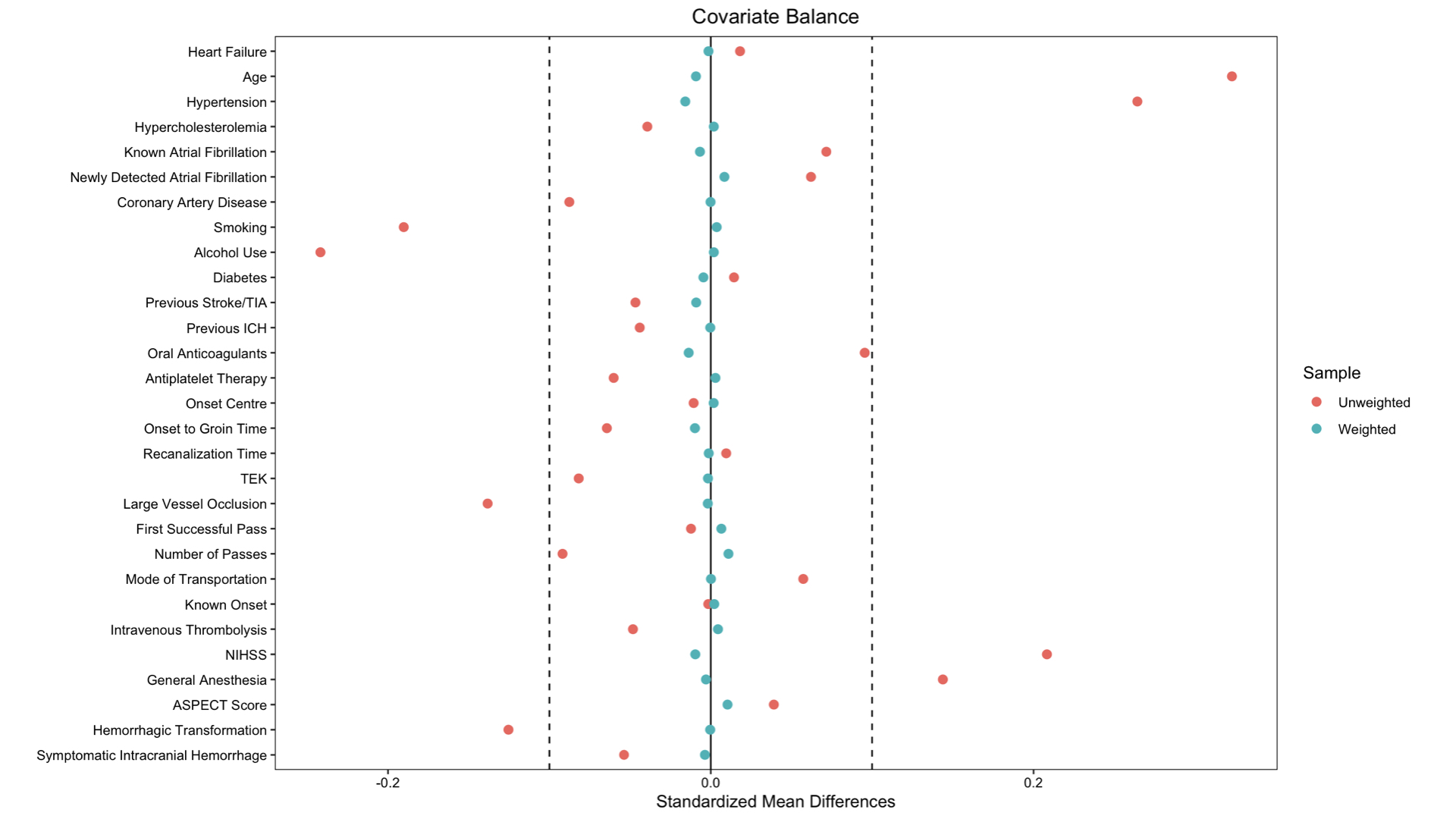

Supplement: Supplementary file 1 — Data S1. [file ENE-32-e70182-s001.docx]
